# Editorial: Unraveling sleep and its disorders using novel analytical approaches, volume II

**DOI:** 10.3389/fnins.2023.1332749

**Published:** 2023-12-06

**Authors:** Gonzalo C. Gutiérrez-Tobal, Leila Kheirandish-Gozal, David Gozal, Roberto Hornero

**Affiliations:** ^1^Biomedical Engineering Group, University of Valladolid, Valladolid, Spain; ^2^Centro de Investigación Biomedica en Red en Bioingeniería, Biomateriales y Nanomedicina, Valladolid, Spain; ^3^Department of Neurology, The University of Missouri School of Medicine, Columbia, MO, United States; ^4^Joan C. Edwards School of Medicine, Marshall University, Huntington, WV, United States

**Keywords:** sleep apnea, automated sleep stages, insomnia, depression, explainable artificial intelligence, GOAL questionnaire

## Introduction

**Figure 1:**
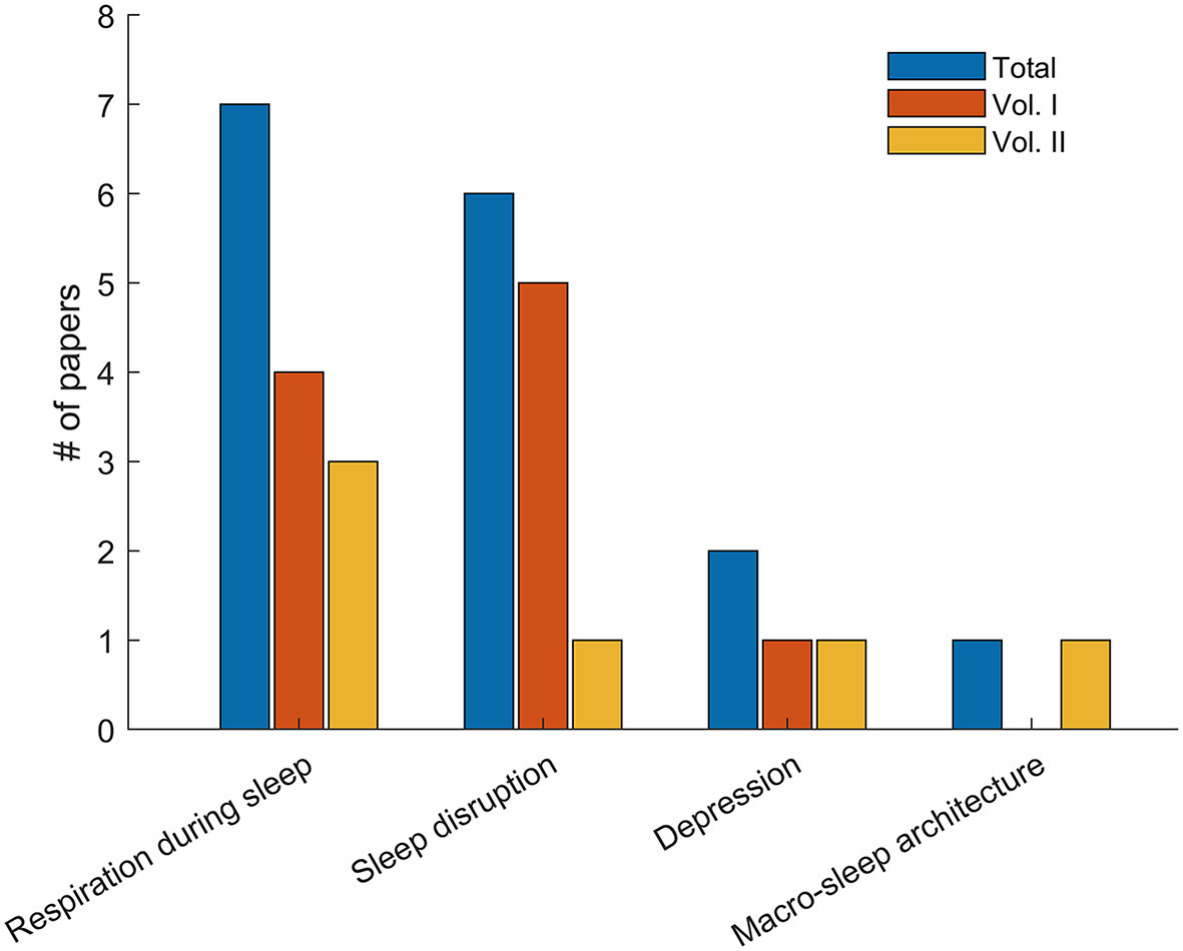
Number of papers published for each topic in Volume I (orange), Volume II (yellow), and the both of them together (blue).

After completion of this second volume, two important aspects have emerged: (i) the manuscripts submitted have met with the highest scientific standards and (ii) a quick comparison with the first Research Topic (Gutiérrez-Tobal et al., 2022) reveals new trends in the application of analytical tools, yet still being applied to unresolved problems in sleep research (see Figure 1). Accordingly, sleep apnea is the subject of investigation in three out of the five studies of this second collection. Novel artificial intelligence approaches focusing on explainability (Adadi and Berrada, 2018), as well as simplified screening procedures based on questionnaires are the topics covered by the authors of these studies. Automatizing sleep stage detection is also a recurrent topic and is present in another paper. Artificial intelligence, and in particular deep learning (Goodfellow et al., 2016), is again the analytical framework selected by the investigators. The fifth article focuses on relationships between insomnia and depression, with analysis of cerebral blood perfusion, as well as the connectivity between brain regions, representing the specific emphasis explored by this investigation.

Consistent with the above-mentioned methods used in the articles, we have organized this editorial in the following sub-sections.

###  Deep learning and explainable artificial intelligence

Deep learning have led to impressive performances in a wide range of problems, including those within healthcare (Miotto et al., 2018) and in the processing of physiological signals (Faust et al., 2018) contexts. Often blamed for being essentially a “black box” methodology, substantial effort is now being made to provide explainable deep-learning models (Adadi and Berrada, 2018). In this Research Topic collection, the study by Pini et al. used the cardio-respiratory signals derived from a wearable chest belt to train a deep learning model with the ability to automatically detect sleep stages. A particular strength of the study is that the investigators included more than 1,000 subjects and evaluated a combination of temporal convolutional (Lea et al., 2017) and inception residual networks (Szegedy et al., 2017) to detect two (wake/sleep), three (wake/NREM/REM), or four (wake/light/deep/REM) sleep stages. Such, sophisticated methodologies clearly open the way for expanded generalizability studies that reconcile different sensors and signals to enable extraction of valid sleep staging irrespective of the specific physiological signal or signals being acquired by the wearable device.

A second study by Serrano Alarcón et al. focused on detecting apneic events during sleep by means of a convolutional neural network (CNN) architecture. In this instance, they used overnight blood oxygen saturation, heart rate, and thoracic and abdominal respiration from more than 4,000 adult subjects from Sleep Health Heart Study (SHHS) and Multi-Ethnic Study of Atherosclerosis (MESA) databases. In their study, the authors also applied explainable artificial intelligence (XAI) techniques such as Gradient-weighted Class Activation Mapping (Grad-CAM) (Selvaraju et al., 2017), which allowed them to visually identify those regions of the signals in which the CNN was focusing to derive its accurate predictions.

###  Brain connectivity and cerebral blood floor

Other issues that remain persistently under exploration in sleep research are insomnia and depression. In this Research Topic, both are addressed at the same time by Xu et al.. The authors presented a very interesting study with 44 patients and 43 healthy controls to pave the way toward finding the mechanisms driving comorbidity of chronic insomnia and major depressive disorders. They showed new connections and associations between cerebral blood flow, brain function, and sleep and emotion regulation abnormalities, which may be behind of the pathogenesis of comorbidity in these diseases.

###  GOAL questionnaire assessment

A new tool in the form of a simple yes/no questionnaire has been recently proposed for sleep apnea screening in adults (Duarte et al., 2020). The GOAL questionnaire (the initials standing for Gender, Obesity, Age, and Loud snoring) showed interesting screening performance when first proposed using a large Brazilian population (*N* = 7,377). In this Research Topic, a first study by Zheng et al. validated the GOAL instrument in a relatively large Asian population from China (*N* = 2,171). The authors reported similar results to those of the original study, as well as comparable screening ability than other well-known questionnaires, thus evidencing its robustness. Furthermore, a second study by Zhao et al. assessed the screening ability of GOAL questionnaire when combined with neck circumference or neck-to-height ratio by the use of logistic regression models. In their study, the authors enrolled 288 subjects and showed improved performance when compared with the original GOAL instrument alone.

## Conclusions

Several conclusions can be derived from the review of the literature that is also reflected in this Research Topic. First, sleep science is a natural multidisciplinary research area. Psychiatrists, electronic engineers, pediatricians, nurses, pulmonologists, radiologists, and computer scientists are among the authors of the published works. Second, studies involving sleep are increasing in the number and magnitude of the participants enrolled in such studies. This was a common drawback in the past due to the low availability of sleep labs. However, social awareness and technical improvements have allowed for the ability to enroll more subjects in these studies, and such enhanced representation has and will continue to improve the quality and significance of the studies being published in these pages. In this Research Topic, three out of the five published works used recordings from more than 1,000 subjects. Finally, artificial intelligence and XAI are gaining importance in the evolution of sleep research, as depicted in two of the published studies. Favored by the above-mentioned increasing in data collection, which is mandatorily required for training successful models, the combination of deep learning and XAI is now providing very accurate methods for the purpose they are designed, while also uncovering new sleep-related knowledge based on the explanations of the decisions automatically made by these models (Vaquerizo-Villar et al., 2023).

## Author contributions

GG-T: Conceptualization, Funding acquisition, Supervision, Writing – original draft, Writing – review & editing. LK-G: Conceptualization, Funding acquisition, Supervision, Writing – original draft, Writing – review & editing. DG: Conceptualization, Funding acquisition, Supervision, Writing – original draft, Writing – review & editing. RH: Conceptualization, Funding acquisition, Supervision, Writing – original draft, Writing – review & editing.
